# Integrating Health and Care in China: Lessons Learned and Future Outlook

**DOI:** 10.5334/ijic.5681

**Published:** 2021-11-08

**Authors:** Linlin Hu, Ye-Fan Wang Glavin, Runnan Yan, Chenyang Pei, Mudan Yan, Yu’ou Zhang, Yuanli Liu

**Affiliations:** 1 School of Health Policy and Management, Peking Union Medical College, Beijing 100730, China; 2 School of Medicine, Case Western Reserve University, Cleveland, Ohio 44106, USA

**Keywords:** integration, health and care policy, China

## Abstract

**Background:**

An aging population is one of the key drivers reshaping health care systems. In China, the complex needs of its huge aging population require integration across the health and care sectors.

**Policies and progress:**

Over the past decade, the central government of China promulgated a series of policies to promote the establishment of aftercare facilities, specify approaches to integrate health and care service delivery at institutional and community levels, pilot long-term care insurance (LTCI) as a funding mechanism, and reform administrative structures in favor of integration. Progress has been made towards organizational and clinical integration of service delivery both at institutional and community levels. LTCI has been introduced as the financing mechanism covering long term care services.

**Discussions and Conclusions:**

The experiences of China in the integration of health and care could be summarized as a top-down approach in policy formulation and implementation, the significant employment of pilots and demonstrations, and the activation of market forces. However, China is still in the initial stage of integrating health and care and is faced with system-level challenges in its financing, management, and workforce, and faces technical challenges, such as a lack of tools, and standards. In the future, these issues need to be addressed.

## Introduction

China is now facing a rapidly aging population. Between 2015 and 2050, the number of people aged 60 years and over will more than double from 222 million to 480 million [1]. In addition to the enormous size of the elderly population, the number of older people with disabilities and chronic disease is significant. There were an estimated 40.6 million older people with disabilities in China in 2015, and this number is expected to reach 96 million in 2050 [2]. Older people are more likely to have chronic diseases such as stroke, cardiovascular disease, diabetes, hypertension, and Alzheimer’s disease or related dementias, and have multiple comorbidities. According to official data, there were 180 million older people with at least one chronic disease in 2018, which was 75% of the total older population [3].

Population aging is one of the key drivers reshaping health care systems all over the world. The presence of multiple disease conditions and physical frailty makes disabled older people complex cases who require treatment and care at various health and care settings and specialties [4]. A holistic approach to integrated care needs to consider not only integration inside the health sector but also the integration of social services [5]. Ensuring the coordination and continuity of health and care is necessary for the elderly as it enhances the quality of care and patient experience and is more cost-effective [6]. However, there are many challenges in care integration, especially between health and care, because the financing, provision, and regulation is usually separated [7].

In China, as in many other countries, health and care have been separated and fragmented. The main regulatory body for social service is the Ministry of Civil Affairs (MCA), while health care is under the supervision of the National Health Commission (NHC). This posed great challenges for the coordination and integration of health and care, resulting in many unmet needs and misallocation of resources. In order to promote care integration, the Chinese government has carried out a series of policies and efforts in the past decade. The aim of this paper is to review the reforms and progress of these efforts, examine lessons learned, challenges remaining, and propose further policy considerations.

## Context

China was formally labelled an aging country in 2000 when the population aged 60 and over reached 10% of the total population [8]. As the country with the largest aging population in the world and now confronting the inability of its traditional bedrock, the family, to care for the elderly, China has embarked on building a formal elderly care system [9]. The central government outlined a plan for a three-tiered elderly care system in 2005, which emphasized home-based care as its foundation, backed by community-based care and supplemented by institutional care [10]. In late 2000s to early 2010s, there was a “leap-forward” development in institutional care, with the total number of residential care beds increasing from 1.54 million to 6.73 million from 2006 to 2015 [11]. Although the demand for nursing home beds is surging, the occupancy rate showed a downswing during this period [12]. This can be attributed to multiple reasons [13], and the lack of professional medical and nursing care is an important one [14]. For example, it was reported that the occupancy rate of elderly care institutions containing medical clinics was over 75% in Beijing at the end of 2016, while the average occupancy rate of those without medical services was less than 30% [15]. The integration of health has become an inevitable choice for elderly care facilities.

In terms of health care, a universal medical insurance system was established in China after the major healthcare reform in 2009 [16] and the demand for medical services has been released. However, due to the lack of availability of aftercare, medically stabilized patients who need rehabilitation or extended nursing care had to remain in hospitals unnecessarily (i.e. social hospital stays) [14], or be re-admitted frequently, leading to the heavy use of the hospital resources and escalating health care costs [17,18]. As a result, in the three-tiered medical system, large tertiary hospitals are often preferred by patients when they are hospitalized, with the bed utilization rate reaching full capacity in 2013 [19]. However, some secondary hospitals had insufficient occupancy because of their limited professional skills and technical support. These are expected to transform into aftercare facilities, such as geriatric hospitals, rehabilitation hospitals, or skilled nursing facilities to receive patients discharged from large hospitals [20].

## Reforms in the past decade

In response to the above issues, the Chinese government has come up with a series of policies since the early 2010s (***Table 1***).

**Table 1:** Main policy documents for integration of health and care.


DOMAIN	POLICY DOCUMENT(S)	DEPARTMENT	TIME	MAIN CONTENTS

Service delivery	Basic Standards for Skilled Nursing Facilities (2011)	MOH	2011.03.21	Stresses the role of skilled nursing facilities as an important part of the health care system; specifies the service contents and standards of skilled nursing facilities

	Basic Standards for Rehabilitation Hospitals (2012)	MOH	2012.04.25	Proposes specific construction standards of Rehabilitation Hospitals

	Several Opinions on Accelerating the Development of Elderly Care Services	State Council	2013.09.06	Encourages medical services to be provided in elder care institutions, communities and families, and requires medical institutions to better provide chronic disease prevention

	Guiding Opinions on Promoting the Integration of Health and Care Services	NHFPC & MCA, etc.	2015.11.18	Defines the goals and proposes the main forms and supporting measures of integration

	Notice on Facilitating the Licensing of Integrated Health and Care Institutions	MCA & NHFPC	2016.04.08	Strengthens intersectoral cooperation to create a “barrier-free” environment for registration of new institutions

	Guidance on Promoting the Family Physician Contracted Services	State Council	2016.05.25	Requires “family physician-led teams” in community health institutions to sign contracts with residents and provide them with services including basic medical care, public health and certain health management services

	Notice on the First Batch of National Level Pilot Units for the Integration of Health and Care Services	NHFPC & MCA	2016.06.16	Selects 50 cities (districts) as the first batch of national level pilot units for the integration of health and care services

	Notice on the Second Batch of National Level Pilot Units for the Integration of Health Care Services	NHFPC & MCA	2016.09.14	Selects 40 cities (districts) as the second batch of national level pilot units for the integration of health and care services

	Basic Standards and Management Standards for Rehabilitation Medical Centers and Nursing Centers	NHFPC	2017.10.30	Encourages private sector to establish community-based rehabilitation and nursing facilities

	Notice on the Replacement of Administrative Approval with Registration for the Establishment of Medical Institutions within Elder Care Institutions	NHFPC	2017.11.08	Cancels the administrative approval required for establishing medical facilities within elder care institutions, to be replaced by registration

Financing	The Plan for Deepening the Reform of the Health Care System during the 12th Five -Year Plan Period	State Council	2012.03.14	Encourages commercial insurance companies to develop long-term care insurance products actively

	The 13th Five-Year Plan for National Economic and Social Development	State Council	2016.03.16	Proposes to “explore building a LTC insurance system” in this period officially

	Guiding Opinions on Launching the Pilot of LTCI System	MHRSS	2016.07.08	Launches LTCI pilots and selects 15 cities across the country as the first batch of national pilot cities

	The 13th Five-Year Plan for the Development of National Elderly Care Cause and Elderly Care System	State Council	2017.02.28	Encourages commercial insurance companies to develop long-term care insurance products and services to meet the diversified and multi-level needs for long-term care of the elderly

	Guiding Opinions on Expanding the Pilots of LTCI System	NHSA etc.	2020.09.16	Expands LTCI pilots to 49 cities


Source: Compiled based on policy documents obtained from *www.gov.cn* and *www.pkulaw.cn*.

### Strengthening the construction of aftercare facilities

Aftercare facilities refer to post-acute or long-term care facilities providing rehabilitation, nursing and some medical support after patients are discharged from acute care hospitals. These have been scarce in China for a long time. As of 2010, there were only 268 rehabilitation hospitals and 49 skilled nursing facilities across China [21], causing a huge gap between supply and demand. To promote the development of these facilities, the current Ministry of Health (MOH) promulgated a policy document of “Basic Standards for Skilled Nursing Facilities” in 2011 [22], stressing the role of skilled nursing facilities as an important part of the health care system to improve the continuity and integrity of care. Skilled nursing facilities were designated to provide patients with medical, nursing, rehabilitation and palliative care, with the aim to care for patients discharged from hospitals with the goal of increasing the efficiency of health care system. In this document, measures such as incorporating the construction of skilled nursing facilities into regional health care planning, transforming existing hospitals into skilled nursing facilities, and encouraging the private sector participation were proposed. Similarly, the “Basic Standards for Rehabilitation Hospitals” was promulgated by the MOH in 2012 [23]. In order to further encourage the private sector to establish community-based rehabilitation and nursing facilities, the “Basic Standards and Management Standards for Rehabilitation Medical Centers and Nursing Centers” was issued by the National Health and Family Planning Commission (NHFPC, renamed from MOH in 2013) in 2017 [24].

### Promote the integration of health and care services

The integration of health and care was first proposed in a policy document to advance the development of elderly care in 2013, encouraging medical services to be provided in elderly care institutions, communities, and home-settings, and require medical institutions to provide better chronic disease prevention, rehabilitation, and nursing care for the elderly [25]. In 2015, the integration of health and care was formally introduced as an independent policy. Nine departments, including the NHFPC and the MCA, jointly issued the “Guiding Opinions on Promoting the Integration of Health and Elderly Care Services”. This policy document defined the goal and proposed the main structure and supporting measures of integration. The basic goal was to set up a policy and regulation system and build a comprehensive and continuous integrated service network for the elderly in both urban and rural regions by 2020. The five forms of integration proposed included: cooperation between health institutions and social care institutions; provision of health care services in social care institutions; encouraging medical institutions to provide social care services; encouraging the private sector to set up institutions of integrated care; and the extension of health care services to communities and families. The first four forms are mainly care integration at the institutional level and the fifth is integration at the community level. Various supporting measures were proposed to support these integration efforts, such as investment and financing, regional planning, workforce development, IT system formation and inter-departmental collaboration.

To simplify and streamline administrative procedures for setting up new facilities providing integrated care, MCA and NHFPC jointly issued a policy to strengthen intersectoral cooperation to create a “barrier-free” environment for the registration of new institutions in 2016 [26]. In 2017, the NHFPC canceled the administrative approval formerly required for establishing medical facilities within social care institutions, to be replaced by routine registration [27].

Care integration at the community level is facilitated by ongoing reform of strengthening of the primary health care system [28], in which the contracted services of family physicians were strongly promoted by the government. In a major policy document in 2016 [29], the government required residents to sign contracts with “family physician-led primary care teams” in community health institutions to receive a package of basic care including medical care, preventive care, and health management (including health assessment, rehabilitation guidance and home-based medical care, etc.). Primary care teams were led by family physicians or general practitioners with the support of nurses and public health professionals at community health centers, which are mostly set up and funded by local governments (municipalities or counties). The service packages provided varied across regions and would be adjusted over time, with cost shared by medical insurance, public health funds and the residents. Following this policy, the “family physician-led primary care teams” are accountable for residents’ overall health and wellbeing, including extending health care services to community care organizations and home-settings to support care integration at the community level.

### Piloting long-term care insurance (LTCI)

Entering the 21^st^ century, China has gradually established a basic social medical insurance system of universal coverage. By the end of 2019, 1.35 billion people in the country were enrolled in basic medical insurance schemes, with the insured rate stabilizing above 95% [30]. The proportion of the elderly enrolled in social medical insurance schemes in urban and rural areas has reached 98.9% and 98.6%, respectively [31]. Medical insurance covers health care only, while long-term social care is mainly financed by private payments supplemented by taxation and lottery funds [32].

By learning from the experiences of countries such as Germany, Japan, and South Korea, China began to explore a LTCI system after 2010. There were some local pilot programs aimed at establishing LTCI in the early 2010s. In 2015, “The 13th Five Year Plan for National Economic and Social Development” officially proposed to “explore building an LTCI system” by 2020. In June 2016, the LTCI demonstrations were launched by the Ministry of Human Resources and Social Security (MHRSS, the ministry in charge of social medical insurance at that time) [33]. Fifteen cities across the country were selected as the first batch of national pilot cities. The goal was to explore a social insurance system that provides funds for “daily personal assistance and skilled nursing care in support of the activity of daily living” (a combination of health and care), mainly for long-term and severely disabled people.

Besides social LTCI, the government also encouraged insurance companies to develop LTCI-related commercial products, starting as early as 2012 [34,35].

### Reform of Governance Structure

Before 2018, the NHFPC was in charge of health care, the MCA was in charge of long-term social care, and the MHRSS and NHFPC were jointly in charge of social medical insurance. The policy of integration of health and care was mainly undertaken by the Family Development Department of the NHFPC in coordination with the MCA, while the management of LTCI was undertaken by the Social Insurance Department of the MHRSS. In the organizational reform of the State Council in 2018, the “Department of Elderly Health” was established for the first time by the NHC (renamed from NHFPC in this reform), integrating the former Office of Aging of the MCA, and was designated to coordinate health policies and develop a health care system for the elderly. Compared with the previous Department of Family Development, the “Department of Elderly Health” has a stronger position in policy coordination and resource integration [36]. In the meantime, the National Healthcare Security Administration (NHSA) was established as an independent agency under the State Council, integrating the management of basic medical insurance schemes and LTCI, which laid an organizational basis for the policy and financial coordination of care integration.

## Progress of Care Integration

### Service delivery

By 2016, a total of 90 city districts/counties had been identified as national pilot sites for the integration of health and care [37,38]. Different models of integration and implementation strategies have been demonstrated, including both institutional and community-based care.

#### Care integration at institutional level

- **Cooperation between health and care institutions**. This was achieved mainly by signing service and referral agreements within these two types of institutions, usually stipulating regular visits and interventions provided by medical institutions for residents in elderly care institutions, and admissions of patients discharged from the medical institutions by elderly care institutions.
- **Incorporation of health care in elder care facilities**. This is usually realized by establishing medical clinics, nursing stations, and rehabilitation or geriatric hospitals in elder care institutions. Medical clinics and nursing stations are often set up in small and medium-sized elder care institutions with full-time medical staff employed as well as part-time physicians with multi-site practices. The health care staffs provide comprehensive medical diagnosis, treatment, and nursing care for the residents who have medical needs. New large-scale aged care communities usually build a hospital within the community. For example, Yan Garden, a large continuum care retirement community established by Taikang Insurance Company, provides all levels of care to independent, partially and severely disabled elderly. In Yan Garden, a rehabilitation hospital was established to provide health promotion, disability prevention, disease management, and rehabilitation for the residents in the community [39].
- **Incorporation of social care in health care facilities**. This involves either establishing skilled nursing units or day care centers in medical institutions, or the transformation of general hospitals into rehabilitation hospitals, geriatric hospitals, or skilled nursing facilities. Some institutions qualified to provide both health and care set up so-called “swing beds”. The nature of the swing beds is determined by the status of the patient as medical or social needs, thus realizing seamless transfer between health and care. Luohu District Hospital Group in Shenzhen City, Guangdong Province is one of the best-known integrated care models in China, integrating 5 hospitals (including 1 geriatric hospital) and 23 community health centers in the district. The providers are paid on a per capita basis by the medical insurance, which created incentives to strengthen community-based primary care management and facilitated vertical integration between acute and primary care. The geriatric hospital serves as an aftercare facility in the group. With licenses as both a health and care institution, it has 104 medical beds and 952 social care beds [40]. In practice, the nature of beds flexibly change between “health care” and “social care” according to the needs of the elderly served.

By the end of 2019, a total of 56.4 thousand pairs of health institutions and social care institutions nationwide had signed cooperation agreements. There were 4,795 institutions qualified to provide both health and care, among which 3,172 were social care institutions that provided health care and 1,623 were health care institutions that provided social care [41]. However, compared to the large quantity of institutions across the country (about 1 million health care institutions [42], 34.3 thousand residential social care institutions, and 64 thousand community social care facilities [43] in China in 2019), the number of institutions with both qualifications or providing integrated care remains a very small number.

#### Care integration at community level

The contracted service provided by family physician-led teams has advanced, and according to official data, 60% of older people across the country signed such contracts by 2017 [44]. The contract, which is strongly pushed by the government, is more like a service agreement than a standard contract between the primary care teams and community residents, specifying the service contents and frequency. The home medical bed is a kind of home-based care providing medical and nursing care for those with chronic diseases and disability at home, and is one of the services in the family physician’s contracted service package under medical insurance hospital reimbursement. Shanghai is currently the city with the largest number of “home medical beds” in China. In 2019, there were approximately 72,000 home medical beds in Shanghai [45]. A total of 95.75% of patients admitted are elderly people over 60 years old, and approximately half are over 80 years old. Most of the patients have some disabilities with diseases such as cardiovascular disease, stroke, chronic bronchitis, advanced tumors, and fractures. Service items include medical rounds, physical examination, basic nursing, rehabilitation, traditional Chinese medicine, medication, and palliative care [46]. Health services have also been extended to cover the elders in community care facilities. These facilities are still in the initial development stage, providing mostly day care with some residential care. Integration of health care has been seen as a necessity by these facilities to align with policy directions and integration is carried out in various forms, such as setting up nursing stations inside [47], collaborating with hospitals, community health institutions, or even village doctors in rural areas [48,49].

#### Establishment of aftercare facilities

Despite the government’s intensions, the number of rehabilitation hospitals and skilled nursing facilities in China has grown at a very slow pace, with only 706 rehabilitation hospitals and 628 skilled nursing facilities across the country in 2019 (***Table 2***). It is argued that the medical insurance’s limited coverage of post-acute care is a main reason for the underdevelopment of aftercare facilities [50].

**Table 2:** Number of rehabilitation hospitals and skilled nursing facilities in China.


	REHABILITATION HOSPITALS		SKILLED NURSING FACILITIES	

		NON-PUBLIC(%)		NON-PUBLIC(%)

**2010**	268	118(44.0)	49	27(55.1)

**2011**	301	155(51.5)	60	38(63.3)

**2012**	322	173(53.7)	75	53(70.7)

**2013**	376	214(56.9)	105	84(80.0)

**2014**	396	230(58.1)	126	104(82.5)

**2015**	453	289(63.8)	168	143(85.1)

**2016**	495	332(67.1)	240	208(86.7)

**2017**	552	400(72.5)	349	312(89.4)

**2018**	637	485(76.1)	477	432(90.6)

**2019**	706	545(77.2)	628	571(90.0)


Source: National Health Commission of China, China Health Statistics Yearbook, 2011–2020.

#### Development of private sector in care integration

The role of the private sector has been emphasized in this field, and the past decade has seen a surge of market forces in both institutional and community care. Over 70% of the rehabilitation hospitals are non-public and the ratio is 90% for skilled nursing facilities (***Table 2***), showing the increasing and dominant role of private sector in this field. Many insurance companies as well as real estate companies begun to operate businesses and build new facilities. Public-private partnerships (PPP) have also been widely adopted. According to the Ministry of Finance, 307 elderly care PPP projects were in progress in August 2017, of which 42.1% were projects integrating health care [51]. There are also internet companies developing businesses in home nursing care or online family physician services, serving a complementary role for home-based care.

### Financing

In 2016, 15 cities were designated as national demonstrations for LTCI schemes. LTCI coverage has expanded gradually and approximately 88.5 million people have participated in the pilot cities as of June 2019. Of them, 426 thousand participants (0.5% of the participants) were receiving LTCI benefits (***Table 3***) with an average benefit amount of about 9,200 RMB (1,193 EUR) a year [52]. In 2020, the number of pilot cities was increased to 49 [53].

**Table 3:** Number of Participants and those receiving benefits in LTCI piloting cities.


	PARTICIPANTS (MILLION)	PARTICIPANTS RECEIVING BENEFITS (THOUSAND)

**2017**	44.0	75

**2018**	63.6	255

2019*	88.5	426


*Data until June 2019. Source: Data for 2017 are from *The pilot of long-term care insurance is progressing smoothly*. Ministry of Human Resources and Social Security. *http://www.mohrss.gov.cn/*; data for 2018 is from *Letter of the Ministry of Civil Affairs in response to the proposal to promote the sound development of China’s old-age services, http://www.mca.gov.cn/article/gk/jytabljggk/zxwyta/201911/20191100021091.shtml*; data for 2019 is from *Letter of the NHSA in response to proposal No. 0934 (Sector of Social Administration No. 091) of the second Session of the 13th National Committee of the CPPCC. http://www.nhsa.gov.cn/art/2019/12/3/art_26_2115.html*.

In the first 15 pioneer cities, the funding for LTCI comes from multiple sources, including the transferring of the surplus of basic medical insurance funds, collecting premiums from individuals and employers, government financial funds, and social endowments (such as welfare lottery funds). Eligibility for benefits has been established mainly for severe disability, with moderate disability and intellectual disability eligible in only a few cities. The benefits are provided mainly in the form of in-kind institutional and home care supplemented by cash allowance in several cities. Services covered include a spectrum of medical, nursing, and daily personal assistance [54,55,56]. With the different eligibility and reimbursement criteria in the 15 pilot cities, it was estimated that the proportion of LTCI spending would range from 0.08% to 1.90% of GDP for China in 2015, and this proportion will increase to 0.28% and 5.90% respectively in 2055 as the aged population continues increasing [57]. Therefore, the eligibility criteria and reimbursement rate should be deliberately designed and calculated to ensure the financial sustainability.

With LTCI, a stable source of funds is provided for long-term care and related health care, which stimulates service supply, relieves pressure and reduces the financial costs of medical care [55]. For example, as one of the first cities to establish LTCI, the number of skilled nursing facilities in Qingdao City of Shandong Province has increased greatly. By the end of 2018, there were a total of 107 nursing facilities providing institutional care, and more than 600 institutions providing home care in Qingdao [58]. The per diem cost for LTCI beneficiaries is 10–20% of the cost of those with similar conditions covered by medical insurance in 2012 in Qingdao, reflecting significant cost savings for both families and government [59].

In addition to current national LTCI demonstrations in 49 cities, new LTCI models are also being developed by commercial insurance companies. According to an investigation, 6 insurance companies in China conducted LTCI business in 2014, with 55 products on sale [60]. However, the scale of commercial LTCI is very small, accounting for less than 3% of the commercial health insurance (544.8 billion RMB or 70.8 billion EUR) in 2018 [61].

## Lessons learned and challenges remaining

### Lessons learned

China has come to recognize that the traditional separate model of health and care cannot meet the needs of the elderly in its advancing aging process. There are three approaches China takes in promoting health and care integration:

- ***The top-down approach***. The policy opinions and overall goals of health and care integration were first announced by the central government: the State Council. Then, policy documents were issued at ministerial level by the NHC, the MCA, and other related ministries, specifying development goals, plans, key tasks, and responsibilities, followed by implementation procedures issued by local governments in the context of its local environment.
- ***The utilization of pilot demonstrations***. Although the concept of health and care integration has been discussed and explored for over 20 years globally, it is still difficult to contextualize the international experience with lessons applicable to addressing local needs. Consequently, the Chinese government chose to explore models through pilot demonstrations. The basic principles and framework were determined by the central government, and the specific models, mechanisms, and tools were determined by the demonstration sites. This incentivized local people to innovate and acquired experiences that have a strong spillover effect. The demonstrations have also become a process of consensus-building with improved public awareness, better community acceptance and enhanced implementation feasibility.
- ***The engagement of private sector***. Facing the great challenge of an aging society, the Chinese government has long realized that the market should play an important role in elderly care. The participation of the private sector is greatly emphasized in the provision of integrated care for the elderly by removing market entry barriers and promoting public-private partnerships.

### Challenges remaining

In spite of progress made, China is still in the early stage of integrating health and care. In general, there are some challenges with regard to financing, management, and administration at the system level, and issues such as a shortage of professionals, a skilled workforce and a lack of assessment tools and quality standards.

- ***Lack of break-through and evaluation in local demonstrations***. Although innovations were encouraged by the central government, there have been few break-through changes in local demonstrations. Most of the efforts stay at the superficial level and hardly touch the deep-rooted problems, such as reform of the current poorly coordinated management system, or creation of innovative financing and payment models. Another weakness is lack of systematic evaluation. In formulating the policies, the central government did not specify clear goals or relevant indicators to assess the impact of integration, and little is known about the advantages and disadvantages of different models [62]. In governments also unleashing regional creativity in integration such as Scotland, local authorities are still required to collect data on common outcome measurements to demonstrate the impacts [63]. In general, China does not have such common measurements identified.
- ***The financing and payment system for health and care was unsound***. The development of a financing and payment system favoring integration is very crucial in China. However, the coverage of LTCI should be further defined. In national policy, LTCI is designed to mainly cover “daily personal assistance and skilled nursing care in support of activity of daily living,” and it is required that “medical, rehabilitation and nursing care that is covered by basic medical insurance or injury insurance should not be covered by LTCI.” [64] However, the definition of “skilled nursing care in support of activity of daily living” is not clear, and the benefits of LTCI and medical insurance are not well allocated or coordinated. Local practices in pilot cities varies greatly, leading to disparities among regions. For example, LTCI covers both medical and social care expenses in some cities, while for others LTCI covers only expenses for social care and leaves medical expenses covered by medical insurance, or even uncovered [15,54,55]. Some social care facilities providing medical care are not qualified for medical insurance due to strict regulations which restrict them from receiving residents with medical needs [51]. In addition, current medical insurance is very limited in reimbursing post-acute care. This makes it difficult to coordinate with LTCI. It has been argued that fragmentation of the two insurance schemes does not provide incentives for providers to pursue better cost-saving outcomes [14]. The inadequate financing and payment policy is becoming a significant barrier for the private providers to enter the market to fully grow their commercial business.
- ***Lack of a mechanism and incentive for integration at the community level***. International experience reveals that an effectively coordinated care system should strengthen community-focused care, using primary care services for overall care management [65]. In China, the government expects community and home-based care to be the main body of elder care, and requires community health workers, such as family physicians/GPs and nurses, to extend health care services to community care facilities and families. However, from the perspective of management, there is no official mechanism at the community level to coordinate resources and services to form a point of contact between health and care sectors. Although GPs/family physicians are expected to be responsible for their patients’ health, they are not required to assist in the patient’s transition to social care settings. Another issue relates to incentive. In China, community health institutions are mainly public institutions and the prices of various services are set by the government at generally low levels, especially for home-based medical and nursing care services. Some services are not even listed in pricing tables. In many regions these services are not covered by basic medical insurance [66]. These factors, combined with an inflexible salary system for staff, result in little motivation for community GPs and nurses to provide home-based medical and nursing care [14,67].
- ***Difficulties in intersectoral coordination***. As above mentioned, the financing and regulation of health and care is still spread across different government departments, such as the NHC, MCA, and NHSA. Despite the division of responsibilities in national policy, the administration, planning, and regulation work among these departments is still fragmented in reality [68]. This “multi-headed management” increases the administrative costs of the facilities and hinders the integration of service delivery [69]. The fragmented IT systems among different sectors and facilities also makes it hard to enable data transfer and care continuity.
- ***Shortage of professionals and workforce***. China has a great shortage of qualified human resources in both service provision and management of integrated health and care. Integrated care for the elderly requires multi-disciplinary professionals to coordinate at the clinical level, which encompass geriatrics, GPs, nurses, physical therapists, nutritionists, counselors, social workers, and nurse assistants. The team is generally led by GPs or nurse practitioners as case coordinators. However, the education system and professional track for these clinical and managerial staff are still in the initial stage in China. The shortage of qualified care providers is huge. It is estimated that at least 13.54 million nurse assistants or *huliyuan* are currently needed in China, while there are less than 0.4 million, most of whom are without formal training [70]. To improve capacity, the government has developed some educational programs to train professional care workers. However, the long training cycle, high work pressure, and low wages with few benefits have led to low enrollment in these programs [62,71].
- ***Lack of unified need assessment tool and quality standards***. Needs assessment is the basis for providing integrated care services. The traditional needs assessment instruments in most of the elder care institutions in China are simple, subjective, and do not reflect the actual need for care [72]. The current assessment tools are mainly based on “the Ability Assessment for Older Adults” issued by the MCA in 2013 and some assessment tools developed by LTCI piloting cities. These tools are basically not comprehensive and do not include clinical items. The grading methods for the level of care are not well developed based on actual resource utilization [73,74,75]. There is currently no national-level comprehensive assessment tool and grading standard for level of care. The lack of unified assessment has made it difficult to allocate resources to match people’s real needs [76] and to measure the quality of care across institutions [77]. Assessment tools should be developed based on a country’s service system and insurance scheme. Much work is needed in China towards this goal.

### Future directions

Since there is much that needs improvement in China’s current health and care system, promoting the integration of health and care is an arduous task. However, China has realized the importance of integration and has identified it as a priority at the policy level. With these policies, the continuous integration and improvement of care will dominate the future development of the aged care system. In the future, the following considerations should be taken to further promote the integration of health and care.

- *Improve the “top-level design” of the system, especially the financing and payment system*. Medical insurance and LTCI should be regarded as continuous schemes with their respective coverage clearly defined and reasonably connected. On this basis, the coverage of LTCI should be further expanded.
- *Build mechanisms at the community level to coordinate care and incentivize the current community health providers*. A care coordination system/mechanism should be established to form a single-entry point for needs assessment and service allocation, especially at the community level. Government-run community health institutions should be given more autonomy and accountability in providing services and setting salaries, enabling them to take a more active role in care integration.
- *Strengthen intersectoral cooperation*. The government could consider establishing a higher-level inter-ministerial coordination mechanism on the basis of the NHC, the MCA, the NHSA, and other departments to promote integration. In the short run, a consistent or unified entry and regulation framework for facilities should be established across the sectors [78]. In the long run, a ministry of “Health and Welfare” could be considered to promote integration within the framework of a single department.
- *Strengthen the training and retaining of professionals*. Professional tracks for positions such as nurse practitioners and care coordinators should be established with well-designed career paths. For senior and middle-level professionals, education programs with geriatric training should be developed in medical colleges or vocational schools [36]. For long-term care workers, more training programs should be developed with government subsidies to attract more participants.
- *Establish a unified and comprehensive assessment tool*. This tool should assess care needs comprehensively and support continuous data collection across facilities and systems. On this basis, the service scope and standards of various institutions could be defined, and a service quality monitor and evaluation system could be established.
- *Strengthen research and data collection to support evidence-based policy evaluation and formulation*. As China progresses with care integration and LTCI pilots, it is necessary to do rigorous independent evaluations before upscaling them [62].

## Conclusion

With much accomplished, China still has a long way to go in the integration of health and care. The Chinese government has formulated and implemented its policies by mobilizing local governments and the private sector through a top-down approach and national demonstrations. Since 2010, a significant amount of work has been done, such as integrated care models implemented both at the institutional and community level and financing mechanism of LTCI established in addition to the existing medical insurance scheme. However, the top-level design of the system, as well as some specific models, tools, and standards still need to be improved or established, and the capacity of both health and care systems need to be enhanced. It is anticipated that China will make further progress in the field with the strong adaptive and administrative capacity of the government and an actively participating society and market.

## Abbreviations

LTCI, long-term care insurance

MCA, Ministry of Civil Affairs

NHC, National Health Commission

MOH, Ministry of Health

NHFPC, National Health and Family Planning Commission

MHRSS, the Ministry of Human Resources and Social Security

NHSA, National Healthcare Security Administration
